# Transmission center and driving factors of hand, foot, and mouth disease in China: A combined analysis

**DOI:** 10.1371/journal.pntd.0008070

**Published:** 2020-03-09

**Authors:** Yi Hu, Lili Xu, Hao Pan, Xun Shi, Yue Chen, Henry Lynn, Shenghua Mao, Huayi Zhang, Hailan Cao, Jun Zhang, Jing Zhang, Shuang Xiao, Jian Hu, Xiande Li, Shenjun Yao, Zhijie Zhang, Genming Zhao

**Affiliations:** 1 Department of Epidemiology and Biostatistics, School of Public Health, Fudan University, Shanghai, China; 2 Key Laboratory of Public Health Safety, Ministry of Education, Shanghai, China; 3 Laboratory for Spatial Analysis and Modeling, School of Public Health, Fudan University, Shanghai, China; 4 Institute for Infectious Disease Control and Prevention, Qinghai Provincial Center for Disease Control and Prevention, Qinghai, China; 5 Shanghai Municipal Center for Disease Control and Prevention, Shanghai, China; 6 Department of Geography, Dartmouth College, Hanover, New Hampshire, United States of America; 7 Department of Epidemiology and Community Medicine, Faculty of Medicine, University of Ottawa, Ontario, Canada; 8 Department of Geography, Shanghai Normal University, Shanghai, China; 9 Key Laboratory of Geographic Information Science, Ministry of Education, East China Normal University, Shanghai, China; 10 School of Geographic Sciences, East China Normal University, Shanghai, China; Universite de Montreal, CANADA

## Abstract

Hand, foot, and mouth disease (HFMD) has become a major public health issue in China. The disease incidence varies substantially over time and across space. To understand the heterogeneity of HFMD transmission, we compare the spatiotemporal dynamics of HFMD in Qinghai and Shanghai by conducting combined analysis of epidemiological, wavelet time series, and mathematical methods to county-level data from 2009 to 2016. We observe hierarchical epidemic waves in Qinghai, emanating from Huangzhong and in Shanghai from Fengxian. Besides population, we also find that the traveling waves are significantly associated with socio-economic and geographical factors. The population mobility also varies between the two regions: long-distance movement in Qinghai and between-neighbor commuting in Shanghai. Our findings provide important evidence for characterizing the heterogeneity of HFMD transmission and for the design and implementation of interventions, such as deploying optimal vaccine and changing local driving factors in the transmission center, to prevent or limit disease spread in these areas.

## Author summary

Hand, foot, and mouth disease (HFMD) has been a concern in Asian regions since the late 1990s. To disclose the transmission mechanism of HFMD and its driving factors, we combined epidemiological, spatial, and mathematical analysis, using HFMD cases in two provinces (Qinghai and Shanghai) of China as an example. We found the phenomenon of traveling waves in the process of HFMD transmission for the first time and identified different types of traveling waves associated with different driving factors. HFMD in Qinghai decline year by year with the characteristics of cross-region population mobility, while epidemics in Shanghai are persistent in the suburban areas with low incidence in the urban areas and neighboring population movement dominates the pattern. Our findings facilitate the understanding of HFMD transmission mechanisms over geographic regions, which are crucial for designing a jointly effective HFMD prevention and control strategy.

## Introduction

Hand, foot, and mouth disease (HFMD) is a common illness mostly seen in children [1] and is caused by a spectrum of pathogens in the enterovirus (EV) family [2]. Under most circumstances, HFMD is self-limiting and resolves within 7–10 days[3], but some patients can rapidly develop neurological and systemic complications that can be fatal, especially in cases associated with the serotype of EV71 [4]. It has been a concern in Asian regions since the late 1990s [5] since outbreaks of the disease have been documented in Malaysia, Japan, Singapore, Vietnam, and Cambodia [6–10]. In China, HFMD has been prevalent since 2007 and there was a sharp rise in incidence since the Chinese Ministry of Health (MOH) listed HFMD as a notifiable Class-C communicable disease on May 2, 2008 [11]. During 2008–2015, approximately 13 million HFMD cases were reported, including 123,261 severe cases and 3,322 deaths in 31 provinces of mainland China [12].

Understanding the spreading dynamics is vital to limiting HFMD and thereby reducing its adverse impact. However, it is difficult to interpret the transmission pattern of HFMD owing to the presence of nonstationarity and nonlinearity in incidence data. Several factors, including climatic and socio-economic factors, are found to influence the pattern of HFMD, which reflects complex interactions among these factors [13–17]. As a result, incidence data of HFMD show strong seasonality, oscillations, and changes in period over time. These all increase the difficulty of characterizing the particular spatio-temporal transmission dynamics of a host-pathogen system. Previous studies mostly focus on the static spatio-temporal pattern of HFMD [15, 16, 18–21], while its transmission mechanism over time and across space have not been discussed and is still unclear. Characterization of the particular spatio-temporal dynamics of a host-pathogen system can be used to illuminate the mechanisms most important in the dynamic system [22].

In this study, we describe the nonstationarity in the period of recurrent epidemics of HFMD, reveal spatio-temporal dynamics of HFMD transmission, explore the role of spatial heterogeneities in the dynamics, and discuss how the transmissions arise in differing physical and socio-economic environments [23].

## Methods

### Ethics statement

According to China’s law on the prevention and treatment of infectious diseases, personal identifiers should be collected for individual cases with diagnosis of a notifiable disease, for the purposes of public health surveillance and response. The National Health and Family Planning Commission of China decided that the collection of individual data for all notifiable diseases, including HFMD, was part of an ongoing public health response and was thus exempt from institutional review board assessment. The co-authors of this paper in Qinghai and Shanghai Provincial Center for Disease Control and Prevention were given access to the surveillance data for the purposes of research. All the individual HFMD data were anonymized by deleting the personal identifiers (such as patient name, parent name, home address, and telephone number) for the purpose of protecting patients’ privacy.

### Study design and study areas

**Fig 1:**
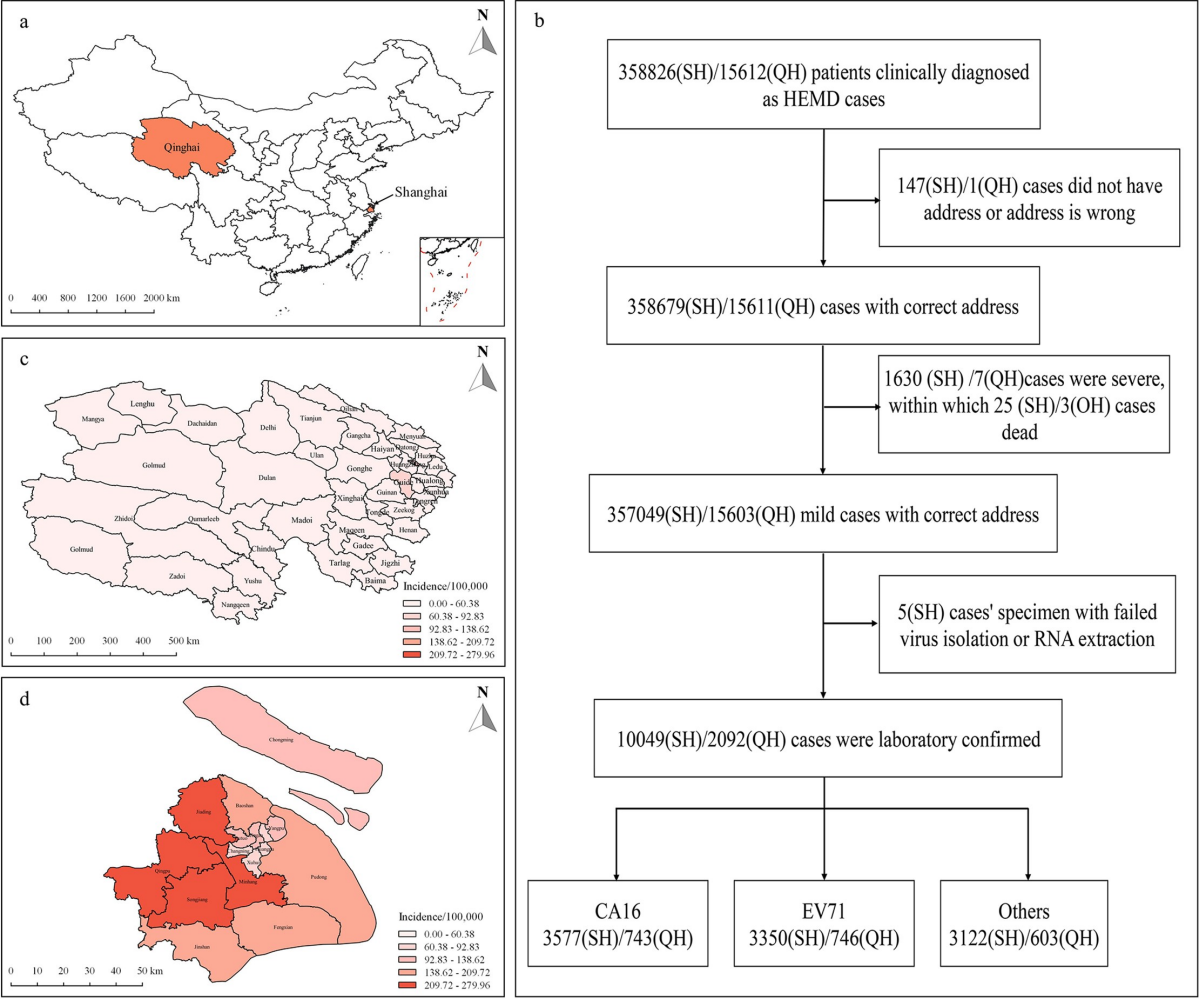
**a. Classification of HFMD based on treatment history and laboratory analysis. b, Location of Qinghai Province and Shanghai Municipality, China. c/d, Annual average incidence of HFMD in Qinghai/Shanghai.** SH = Shanghai; QH = Qinghai. The map a, c, and d were created using ArcGIS software (version 10.4.1, ESRI Inc. Redlands, CA).

We performed a population-based study of HFMD cases in all residents of Qinghai Province (sparse population) with 46 Counties from Western China and Shanghai Municipality (dense population) with 16 Districts in Eastern China (Fig 1A) from 1 January 2009 to 31 December 2016.

Qinghai, located on the northeastern part of the Tibetan Plateau with an average elevation of over 3,000 meters above sea level, spans approximately 721,000 km^2^ in Western China and has a population of 5.98 million (2017) composed of a number of ethnic groups including the Han, Tibetans, Hui, Tu, Mongols, Salars, and several others. Qinghai is administratively divided into eight prefecture-level divisions of which six are autonomous; in turn these are subdivided into 46 counties. Due to the high altitude, Qinghai has quite cold winters (harsh at the highest elevations), mild summers, and a large diurnal temperature variation. Its mean annual temperature is approximately -5 to 8°C, with January temperatures ranging from -18 to -7°C and July temperatures ranging from 15 to 21°C. Significant rainfall occurs mainly in summer, while precipitation is very low in winter and spring, and is generally low enough to keep much of the province semi-arid or arid.

Shanghai lies on China's eastern coast with an average elevation of 4 meters and spans about 6,341 km^2^. As a provincial-level municipality, Shanghai is one of the most populous cities in the world with a population of 24.18 million (2017) composed primarily of the Han ethnic group. Urban areas include seven counties (Huangpu, Xuhui, Changning, Jing’an, Putuo, Hongkou, and Yangpu) and the suburban include nine counties (Minhang, Baoshan, Jiading, Pudong, Jinshan, Songjiang, Qingpu, Fengxian, and Chongming). Shanghai has a humid subtropical climate and experiences four distinct seasons. Winters are chilly and damp whereas summers are hot and humid, with an average of 8.7 days exceeding 35°C annually. The city’s temperature averages 4.2°C in January and 27.9°C in July, with an annual mean of 16.1°C.

### Epidemiological data

Clinically-diagnosed HFMD cases from all medical institutions (e.g. community health-care centers, clinics, hospitals) are statutorily notifiable and reported to Qinghai/Shanghai Center for Disease Control and Prevention (see elsewhere for details on case definitions [1]). Demographic information (sex, date of birth, and residential address), case classification (clinically diagnosed or laboratory confirmed), severity (mild or severe), death status, date of symptom onset and diagnosis, and virus serotype (CA16, EV71, other enterovirus) were collected for all cases using a standardized questionnaire. Individual-level cases were used for epidemiological analysis and the county-based aggregated weekly mild HFMD cases for the spatial and mathematical analyses.

### Socio-economic, geographical, and demographic data

Gross domestic production (GDP), birth population, altitude and density of county-level road, province-level highway, hospital, health workers, and primary and middle schools were collected. Altitude was obtained from an interpolated Digital Elevation Model (DEM) from the Global Land Information System (GLIS) of the United States Geological Survey (http://egsc.usgs.gov/isb/pubs/factsheets/fs06994.html). The remaining data were obtained from the Bureau of Statistics of Qinghai and Shanghai, respectively. Collinearity was investigated between all possible pairs of factors and if any pair had a correlation coefficient >|0.4|, the member of the pair believed less likely to be biologically important was excluded. The finally included variables are density of county-level road, GDP, density of hospital, birth population, and altitude. In addition, we use a temporal basis functions (i.e. natural splines with 3 degrees of freedom) to account for temporal variability in the HFMD incidence data.

### Statistical analysis

We first summarize the basic demographic characteristics of HFMD cases and assess the geographical distribution of HFMD incidence for Qinghai and Shanghai, respectively.

### Wavelet time series analysis

Then, to analyze the periodicity of serotype-specific HFMD cases, we conduct wavelet time series analyses on weekly reconstructed HFMD cases using the Morlet wavelet function [24]. All series are log-transformed first and then scaled to have zero mean and unit variance. To minimize biases due to edge effects, we pad all series with excess zeros. The dynamic patterns of HFMD incidence are then identified by calculating the pairwise phase difference and coherence [25] (which refer to the relative time of peaks and troughs in incidence of geographically disjunct populations) and spatial synchrony [26] (which refers to coincident changes in incidence of geographically disjunct populations) between county pairs in each region.

The pairwise phase difference is used to determine the difference (in weeks) in epidemic timing between counties and it equals the difference of individual phases, Phase.x–Phase.y, when converted to an angle in the interval [−*π*,*π*] (*π* denotes 26 weeks in this study). An absolute value less (larger) than $\frac{\pi }{2}$ indicates that the two series move in phase (anti-phase, respectively) referring to the instantaneous time as the temporal origin and at the period in question (we use 1-year periodicity here), while the sign of the phase difference shows which series is the leading one in this relationship. When in phase, the interval $\left[0,\frac{\pi }{2}\right]$ means x leads y while $\left[-\frac{\pi }{2},0\right]$ means x lags y. We defined a traveling wave as an increasing phase difference with increasing distance as described previously [25, 27, 28]. The pairwise phase coherence of HFMD incidence was defined as the Pearson correlation coefficient of phase angles of each series [25], which is a function of distance separating the geographic centroids of two counties. Spatial synchrony of two HFMD incidence time series was quantified as the Pearson correlation coefficient of those series. Both phase coherence and spatial synchrony were estimated nonparametrically using a spline covariance function [29]. A cubic B-spline of seven and four equivalent degrees of freedom were chosen for Qinghai Province and Shanghai respectively (the square root of the number of counties was used as a guide [29]). We computed the confidence envelopes for the two functions using 500 bootstrap iterations.

### Mathematical analysis

A stochastic spatial susceptible-exposed-infectious-recovered (SEIR) model with Approximate Bayesian Computation (ABC) [30] as the statistical inference method is finally fitted to analyze the transmission mechanism with potential driving factors and population mobility among adjacent areas considered [23], which are captured in the exposure process (i.e., the S to E transition):

$$\pi_{ij}^{SE}=1-exp({\left\{-\eta_{i.}-\sum_{z=1}^{Z}\rho_{z}(D_{z}\eta_{i.})\right\}}^{h_{i}})$$ (1)

where $\pi_{ij}^{SE}$ denotes transition probability from S to E, $\eta_{i.}=\{\delta_{i1}e^{\theta_{i1}},\ldots ,\delta_{in}e^{\theta_{in}}\}$ (*δ*_*ij*_ and *θ*_*ij*_ denote the proportion of persons who are infectious and the exposure intensity parameter at time *i* in spatial unit *j*, respectively), *D*_*z*_ is the *n*×*n* “distance” matrix (where *n* is the number of spatial sites and *z* refers to the number of matrixes which characterize different spatial structures, e.g., neighboring mobility or distant mobility), *ρ*_*z*_ is an autocorrelation parameter subject to $\sum_{z=1}^{Z}\rho_{z}\leq 1$ and {0≤*ρ*_*z*_<1:*z* = 1,…,*Z*}, and *h*_*i*_ is the temporal offset capturing the relative length of continuous time over which the events are accumulated. The exposure intensity parameter *θ*_*ij*_ is represented by a linear function, *θ*_*ij*_ = *X*_*ij*_*β*, where *X*_*ij*_ represents any covariates affecting the exposure process, such as demographic effects, intervention summaries and other spatio-temporal variables, and *β* is the corresponding coefficient. This spatial stochastic SEIR model is a derivative of the metapopulation model, in which the heterogeneity of individual distribution between spatial units (i.e., county) are assumed and individuals are homogeneously mixed within spatial units. Details of this model can be seen in S1 Text.

For the distance matrix *D*_*z*_, we specify two types: a conditionally auto-regressive (CAR) [31] and a gravity matrix, to capture the neighboring movement and cross-region movement of flows of individuals respectively. For comparative purposes, we also consider non-spatial transmission models that assume no flows of individuals among locations. Hence, we build six models, including four non-spatial models (M1: only intercept, M2: intercept + covariates, M3: intercept + temporal basis, M4: intercept + covariates + temporal basis) and two spatial models (M5: intercept + covariates + temporal basis + CAR component, M6: intercept + covariates + temporal basis + gravity component), to illustrate complexities of different exposure processes, which are evaluated by approximate Bayes factors[32]. Finally, posterior predictive distributions of HFMD cases are plotted for each county using the best model and Empirically Adjusted Reproductive Number (EA-RN) [33] trends are used to estimate the reproductive characteristics of HFMD (Details about EA-RN can be seen in S1 Text). Further, if the transmission pattern was best captured with the gravity component, then the network of incoming and outgoing infected individuals constructed by using time-series SIR model [34] (details about this model can be seen in S1 Text) is generated and plotted, in which a dispersion distribution (*m*_*i*,*j*_) are defined as follows:

$$m_{i,j}=\frac{N_{j}^{\alpha }D_{i,j}^{-\delta }}{\sum_{j\neq i}N_{j}^{\alpha }D_{i,j}^{-\delta }}$$ (2)

where *m*_*i*,*j*_ distributes the migrants from county *i* to county *j*, *N*_*j*_ is the population size in county *j*, *D*_*i*,*j*_ is the distance between counties *i* and *j*, and *α* and *δ* denote the dependent effects.

The wavelet time series analyses are implemented using the WaveletComp package of R software (R Development Core Team 2013), and the model fitting of the stochastic spatial SEIR model based on Sequential Monte Carlo-ABC algorithm [35] (Details can be seen in S1 Text) is performed using the R-INLA package.

## Results

### Epidemiological analysis

During the study period, 15603/357049 mild cases of HFMD were reported to the Qinghai/Shanghai CDC surveillance system, in which 2092 (13.41%) /10049 (2.81%) were laboratory confirmed cases (Fig 1B) and the cases from the two regions share similar epidemiological characteristics (Table 1). The median time from illness onset to diagnosis is both 1 day and HFMD occurred more frequently in children under 3 years, male, and scattered children. However, their incidence is quite different. In Qinghai, the annual average HFMD incidence is low (<60.38 per 100,000) in most areas except Xining (the capital city) and Guide (60.38–92.83 per 100,000, Fig 1C), whereas in Shanghai it is higher in the suburban area (>92.83 per 100,000) than in the urban area (< = 92.83 per 100,000, Fig 1D).

**Table 1:** Characteristics of all cases of hand, foot, and mouth disease in Qinghai and Shanghai, 2009–16.

	Qinghai	Shanghai
Age Category (%)		
<3 years	7986 (51.182)	182276 (51.051)
3–4 years	5067 (32.475)	116190 (32.542)
≥5 years	2550 (16.343)	58582 (16.407)
Gender (%)		
Male	9578 (61.386)	215513 (60.360)
Female	6025 (38.614)	141536 (39.640)
Occupation (%)		
Home care children	8718 (55.874)	201593 (56.461)
Preschool children	5783 (37.063)	131965 (36.960)
Students	1036 (6.640)	20968 (5.873)
Others	66 (0.423)	2523 (0.707)
Time from onset to diagnosis(days) (median [IQR])	1.000 [0.000, 2.000]	1.000 [0.000, 2.000]
Laboratory result (%)		
CA16	743 (35.516)	3577 (35.596)
EV71	746 (35.660)	3350 (33.337)
Other EVs	603 (28.824)	3122 (31.068)

Home care children refer to children who do not go to nursery, kindergarten, and primary school; student includes those in primary school, high school, and college/university; others are composed of adult cases.

### Wavelet time series analysis

**Fig 2:**
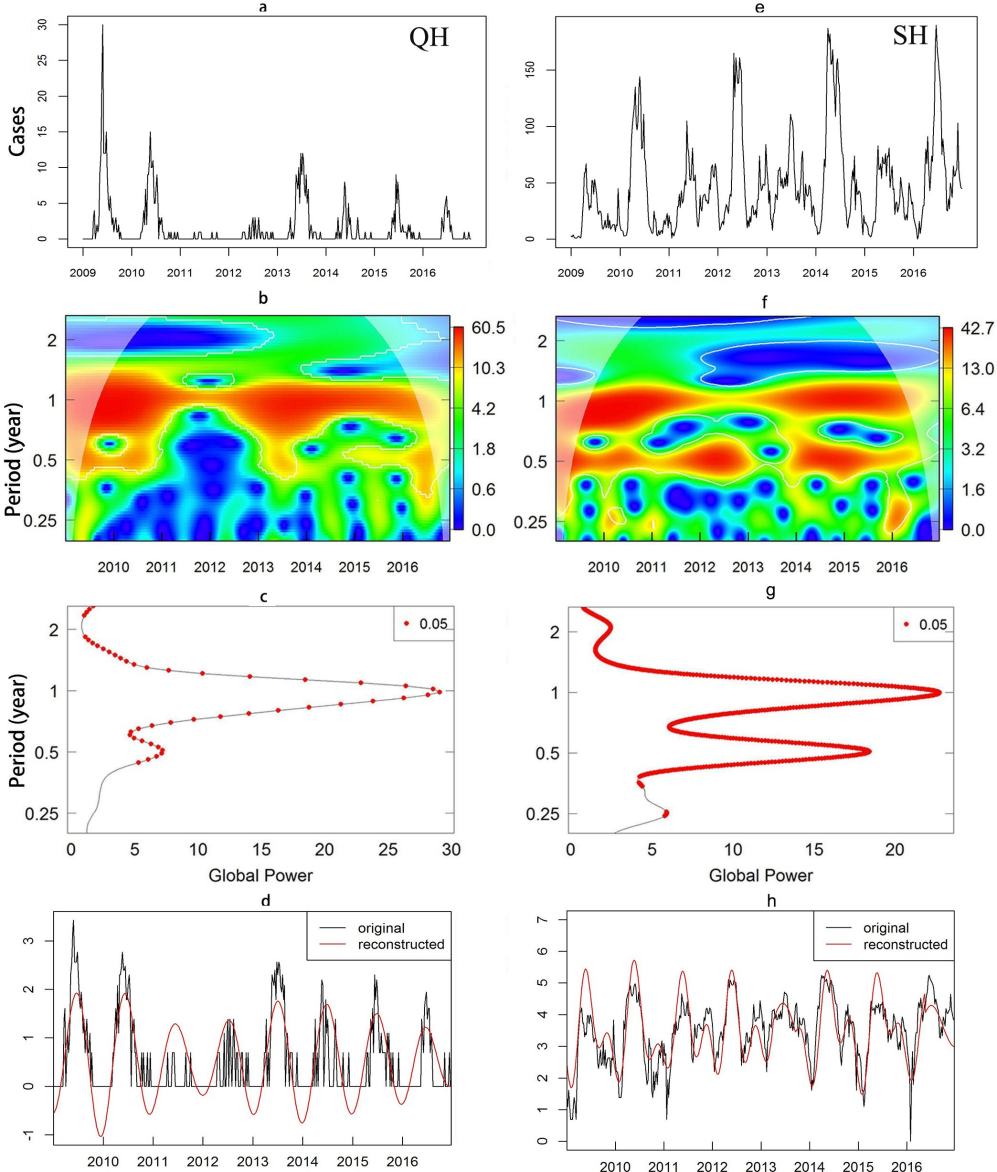
Temporal pattern and periodicity of HFMD. **a/e**. Weekly pattern of HFMD in Huangzhong (Qinghai) and Fengxian (Shanghai). **b/f**, Local wavelet power spectrum in Huangzhong and Fengxian. **c/g**, Global wavelet spectrum (red points represent periods with 95% statistical significance) in Huangzhong and Fengxian. **d/h**, Major epidemic component of the incidence series in Huangzhong (mainly annual) and Fengxian (mainly annual and semi-annual), reconstructed with wavelet spectral analysis from the components in their respective period. All the plots were created using R software (R Development Core Team 2013).

We select one county in each region to show results of wavelet analysis. In Huangzhong of Qinghai, both local (Fig 2B) and global (Fig 2C) powers are the largest at the period of 1 year, suggesting that HFMD cases show a major annual periodicity (i.e., the continuous red region at period of one year in both maps). However, the local maximum power value at the period of 1 and 0.5 years in Fig 2F and Fig 2G suggests that HFMD cases in Fengxian show a major epidemic of annual periodicity combined with an intermittent semi-annual cycle. The reconstructed curves (Fig 2D and 2H), which show a satisfactory overall agreement with the raw data, confirm the correctness of the identified major epidemic period. Results for all other counties (S1 Fig) indicate that there are only 11 counties showing persistent major annual epidemics in Qinghai during the study period while the rest showing sporadic outbreaks, and all counties in Shanghai (S2 Fig) show persistent major annual epidemics.

**Fig 3:**
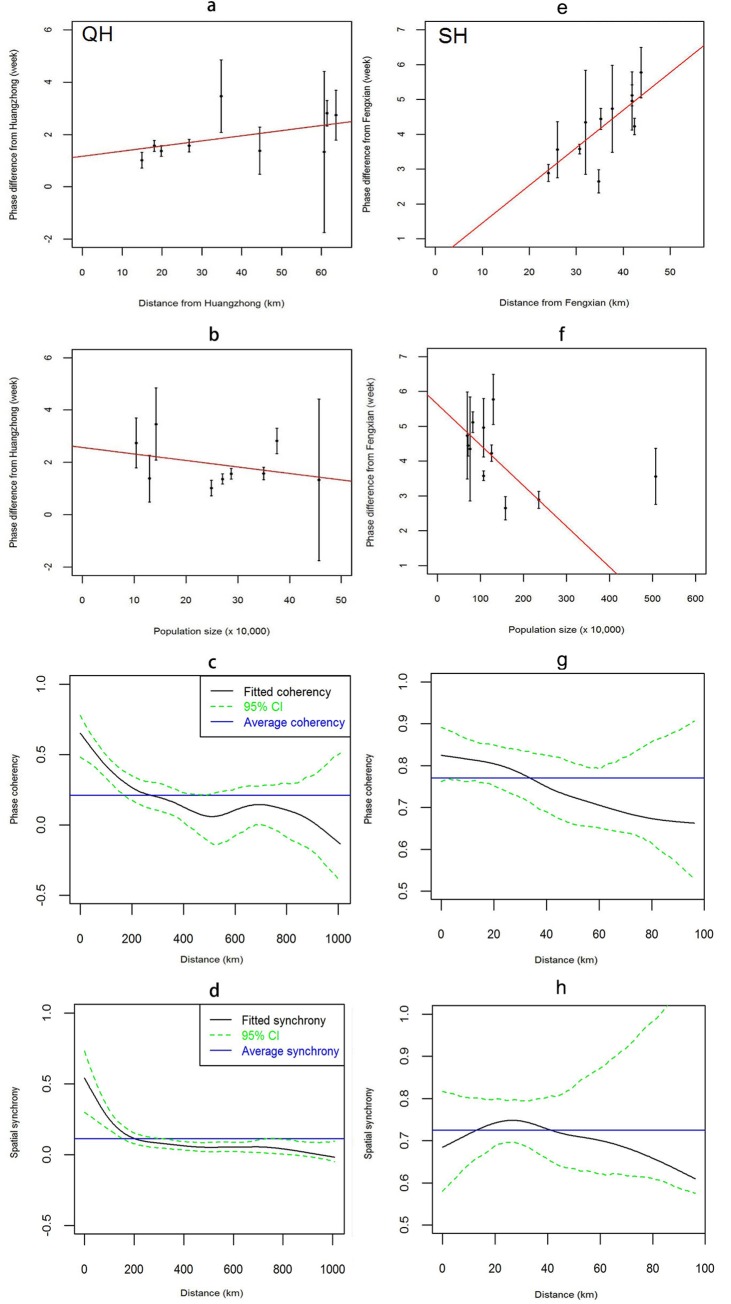
Phase difference, spatial synchrony, and phase coherence plots. **a/e**. Mean phase difference from Huangzhong and Fengxian (as a function of distance from the county). Within 70km of Huangzhong and 50 km of Fengxian, there is an increasing trend in the phase difference with increasing distance (*r*_HZ_ = 0.456, *r*_FX_ = 0.760). The error bars are standard deviation from mean phase difference. **b/f**, Phase difference from Huangzhong and Fengxian (as a function of population size for neighboring counties, within 70 km of Huangzhong and 50 km of Fengxian). There is a decreasing trend of phase difference with increasing population size (*r*_HZ_ = -0.350, *r*_FX_ = -0.611). The error bars are standard deviation from mean phase difference. **c/g**, Phase coherency curve (as a function of distance) of HFMD in Qinghai and Shanghai with 95% confidence limits. **d/h**, Spatial synchrony curve (as a function of distance) of HFMD with 95% confidence limits in Qinghai and Shanghai. All the plots were created using R software (R Development Core Team 2013).

Phase difference analysis reveals that epidemics in Huangzhong and its neighboring counties were “in phase”, with Huangzhong subsequently leading the epidemics in surrounding areas (Fig 3A). The same phenomenon can be seen from Fengxian and its neighboring counties in Shanghai (Fig 3E). The local population size plays a role in these epidemic waves, that is, counties with a smaller population size lag more than ones with larger populations (Fig 3B and 3F); this role is more evident in Shanghai (with more extreme slopes). Phases in nearby locations are generally found to be highly correlated. The correlation declines with distance to yield an average phase coherence of around 210 km (Fig 3C), which can be taken as a rough reflection of the spatial extent of the two dominant travelling waves in Qinghai. The pattern of spatial synchrony in HFMD matches the trends in phase coherence, declining with distance. The case is a little different in Shanghai. The phase coherence (Fig 3G) declines over distance as with in Qinghai while the local spatial synchrony (Fig 3H) fluctuates which is not significantly different from the regional average. In addition, the regional averages (>0.70) of phase coherence and spatial synchrony of Shanghai is much larger than those (<0.25) of Qinghai.

According to approximate Bayes factors, the model M6 and M5 fits HFMD data best in Qinghai and Shanghai, respectively (S1 and S2 Tables). HFMD in Qinghai significantly decreases with increasing GDP, birth population, and altitude with all covariates being significantly associated with infection in Shanghai (Table 2). Significant temporal coefficients suggest infection risk changes temporally in the two regions. The spatial dependence parameter *ρ* shows a higher cross-county spread in Qinghai (0.71) than in Shanghai (0.24). The posterior mean latent and infectious periods 1/*γ*_*EI*_ (0.45 weeks) and 1/*γ*_*IR*_ (1.33 weeks) in Qinghai are acceptable considering that the incubation period of HFMD is 3–5 days and the infectious period is about 7–10 days. Similar values for these two parameters are found in Shanghai.

**Table 2:** Posterior estimates (mean and quantiles) for HFMD in Qinghai and Shanghai, 2009 to 2016.

Parameter	Qinghai			Shanghai		
	Mean	*Q*_0.025_	*Q*_0.975_	Mean	*Q*_0.025_	*Q*_0.975_
density of county-level road	-0.006	-0.021	0.005	**0.124**	0.115	0.136
density of hospital	-0.005	-0.023	0.007	**-0.310**	-0.335	-0.288
GDP	**-0.051**	-0.078	-0.017	**0.081**	0.075	0.089
Birth population	**-0.195**	-0.259	-0.139	**-0.170**	-0.178	-0.159
Altitude	**-0.169**	-0.234	-0.082	**-0.035**	-0.038	-0.032
Temporal 1	**-0.642**	-0.802	-0.483	**-0.190**	-0.220	-0.169
Temporal 2	**-0.266**	-0.410	-0.104	0.041	-0.046	0.143
Temporal 3	**-0.486**	-0.879	-0.210	0.118	-0.009	0.240
*ρ*	0.710	0.558	0.909	0.239	0.232	0.251
*γ*_*EI*_	2.188	1.614	2.837	2.258	1.993	2.661
*γ*_*IR*_	0.751	0.654	0.847	0.763	0.743	0.783

HFMD cases in Qinghai were fitted using model M6 which refers to the model with components of intercept + covariates + temporal basis + Gravity; HFMD cases in Shanghai were fitted using model M5 which refers to the model with components of intercept + covariates + temporal basis + CAR.

**Fig 4:**
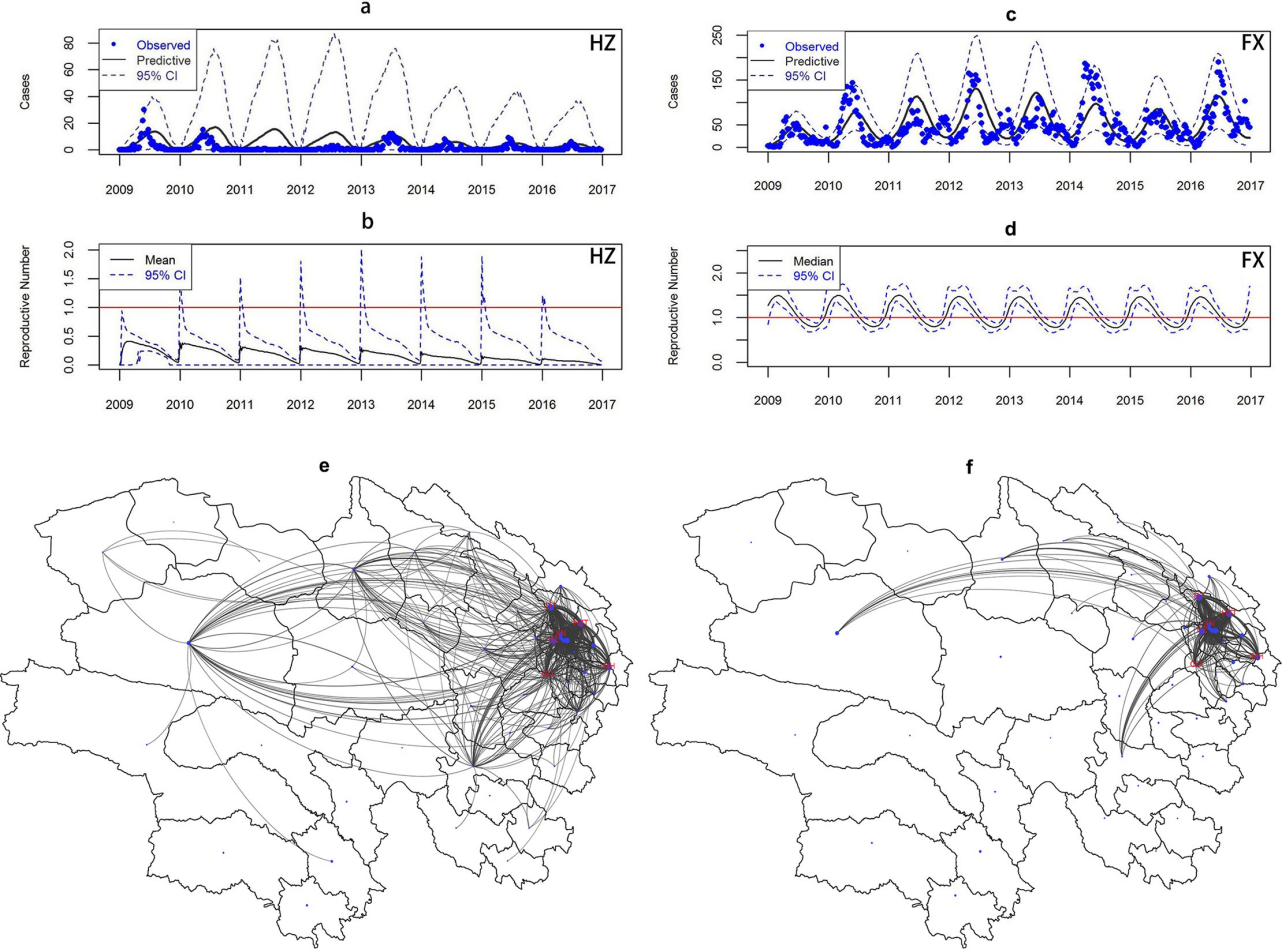
Spatial SEIR fitting results of HFMD transmission. a/c. Posterior predictive distribution for HFMD in Huangzhong and Fengxian. **b/d**, Empirical adjusted reproductive number of HFMD for Huangzhang and Fengxian. **e/f**, network of outgoing/incoming infected individuals in Qinghai. HZ = Huangzhong; FX = Fengxian. The map e and f were created using R software (R Development Core Team 2013).

The posterior predictive distribution demonstrates an acceptable fit in Huangzhong (Fig 4A) and much more reasonable fits are seen in Fengxian (Fig 4C). The EA-RN curves show obvious seasonality in both regions. The reproduction number in Huangzhong is always below one and exhibits both a repeating annual cycle as well as an overall decreasing trend (Fig 4B). In Fengxian, the EA-RN curves reveal repeating cycles that lie above one in the first half-year and below one in the second half-year (Fig 4D). Similar results can be seen in other counties in Qinghai and Shanghai (S3–S6 Figs). The edges of transmission network in Qinghai radiating from the populated counties to reach the small ones (Fig 4E and 4F), potentially causing a reintroduction of the infection in these locations. This link between big and small counties does not seem to depend on geographic distances, while the amount of incoming infections is mostly dependent on distances between locations (Fig 4F) since its edges connecting different counties are shorter than that in Fig 4E. In addition, the parameter estimates for the dispersion term is shown in S3 Table.

## Discussion

The (static) spatial and spatio-temporal pattern of HFMD and potential physical and socio-economic factors associated with the disease have been previously studied, but risk factors and population mobility are seldom considered in dynamics of HFMD. In our study, we employed wavelet time series analysis to characterize temporal dynamics of HFMD and modeled the disease transmission using an SEIR framework to investigate the spatio-temporal heterogeneity in two typical regions of Western and Eastern China. This is the first study, to our knowledge, that finds the phenomena of travelling waves for HFMD (i.e., the disease wave emanated from a center county and moved radially in each region).

Qinghai with sparsely population from Western China and Shanghai with densely population from Eastern China have marked geographic differences and different HFMD incidence, but their epidemiological characteristics of HFMD cases are largely similar (Table 1). The periodicity of HFMD is also different for Qinghai (period of one year) and Shanghai (annual and intermittent semi-annual periodicity), which is consistent with the general view of one annual peak (June) in Northern China and two peaks (May and September-October) in Southern China [1]. The pairwise phase difference analyses shows that Huangzhong and Fengxian lead all counties’ incidence pattern in Qinghai and Shanghai, respectively (i.e., all counties in Qinghai and Shanghai lag behind Huangzhong’s and Fengxian’s incidence pattern, respectively). Huangzhong and Fengxiang are therefore expected to play central role in dynamic of HFMD in respective region. Specifically, in Qinghai, as distance from Huangzhong increases, the phase difference expands (Fig 3A) which indicates that the time shifting of HFMD incidence at peaks (or troughs) becomes larger with increasing distance, and the degree of phase coherence declines which suggests that synchrony of HFMD incidence are present at nearby locations (Fig 3C), demonstrating the presence of spatial waves [36, 37] away from Huangzhong. The wave was particularly well defined up to 70 km from Huangzhong (Fig 3A) with a wave speed of around 31 km per week. Similarly, a repeating travelling wave emanates from Fengxian at a speed of around 11 km per week (Fig 3E).

We further find that the phase difference decreases as peripheral populations increase in size (Fig 3F) and a closer examination on population size over all counties in Qinghai reveals that Huangzhong has the largest population with the exception of Mangya (as shown in Fig 1C, a county located in corner of the northwest with few HFMD cases in the northwest) while all its nearby counties are relatively small, in which persistent epidemics or sporadic outbreaks occurred. This is in line with hierarchical epidemic waves present in measles [25] and dengue [27]. Compared to Qinghai, traveling waves of HFMD emanating from Fengxian in Shanghai occurred among counties that have large populations and persistent major epidemics. The approximation of local spatial synchrony to the regional average with distance (Fig 3H) and the large regional average (>0.70, shown by the blue line) indicate that spatial clustering of epidemics occur over the whole region.

Besides population, we further identified other factors that might affect the traveling waves using a spatial mechanistic model for the spread of HFMD. Qinghai and Shanghai have different significant factors. In particular, GDP had opposite effects in the two regions (negative in Qinghai but positive in Shanghai). A possible explanation for this difference is that Qinghai is a relatively underdeveloped region within which economic inequality is large [38], thus the wealthier families have better health resources and higher consciousness about the disease. Shanghai, however, is one of the most developed regions in China [38, 39]; wealthier families tend to have children participating in extracurricular classes, increasing the infection risk. In addition, the density of county-level roads and the density of hospitals may have significant impacts on infection risk in Shanghai. Denser county-level roads might indicate a higher exposure to the infected individuals and denser hospitals might suggest better health resources. In Qinghai, however, the two factors are not significantly associated with infection risk, which might be explained by imperfect and comparatively limited traffic infrastructure and medical resources. A temporal basis function, included in the exposure process to account for temporal variability of HFMD cases due to seasonality and socio-economic changes [23] not captured by our covariates, is validated by the Bayes factor results favoring the model with this function. The differences in the three temporal basis parameters between Qinghai and Shanghai reflects a difference in the residual temporal trend of HFMD incidence.

We also found that the pattern of population mobility varies between the two regions with results of model comparison (S1 and S2 Tables). In Qinghai, the population tends to move larger distances across the region which indicates that both close and distant HFMD transmission occur and large counties play an important role in starting outbreaks in smaller counties in this sparsely populated region (Fig 4E and 4F). The finding that individuals tend to move long distance is supported by their life style. The minority nationalities accounted for about half of the population in Qinghai, who live in large and sparsely-populated regions and work on agricultural and animal husbandry production [40]. Those in Shanghai tend to move between neighboring areas and the neighborhoods’ contributions to epidemics in one county should be responsible for the travelling waves in this densely populated region. Together with the two centers (i.e., Huangzhong and Fengxian) for the travelling waves, the pattern of population mobility suggests that Huangzhong and Fengxian are the priority areas for targeted interventions in the respective region.

For the instant change of infectious behavior of HFMD pathogens, the overall decreasing trend of EA-RN in Huangzhong suggests an outbreak of HFMD declines over time and would eventually die out. The same profile is seen in most other counties in Qinghai (S5 Fig). Nevertheless, in Fengxian, EA-RN trend indicates the disease repeatedly declines from epidemic during the study period. Counties in suburban areas have similar profile with Fengxian, but most of those in urban areas experience constant low incidence, suggesting heterogeneity in transmission behavior in Shanghai. Note that the disease still exists although EA-RN is below one over time in Qinghai. This might indicate that, in addition to direct transmission via human contact, indirect transmission via free-living viruses from the environment exists. In the environment, the pathogen EV-71 can survive for a long period in suitable condition outside the host [41]. Hence, individuals can get infected through contact with contaminated environment such as water, food, or surfaces [42]. In addition to contaminated environments, asymptomatic individuals plays an important role in epidemic outbreaks [43, 44]. The two factors cannot be accounted for when calculating EA-RN.

The reproductive number (i.e., EA-RN) used in our analysis is largely different from those used in previous studies which typically make use of the basic reproductive number (*R*_0_) or the effective reproductive number (*R*_*e*_). EA-RN takes into account all temporal and spatial factors that affect the exposure process and thus reflects the instant change of these factors on the infectious behavior of a disease. Further, EA-RN considers the nonlinear effect on the contact rate of the number of infectious individuals and is capable of overcoming under-specification of the exposure process (i.e., when some exposure factors are unknown or not available) [33]. These issues cannot be addressed by the traditional *R*_0_ or *R*_*e*_. Therefore, we believe EA-RN is more suitable for epidemiological studies focused on disease control and prevention and should be recommended for use in the future.

A number of limitations of our study deserve discussion. First, we were not able to fully compare the spatio-temporal dynamics of specific virus serotype of HFMD cases in the two regions given the limited number of HFMD cases in Qinghai. Second, we used a basis function to account for the temporal variation of the disease incidence but interpretation of the three temporal basis parameters in Table 2 is not so straightforward; in this regard, consideration of climatic factor and socio-economic changes as should be warranted in a future study. Finally, we faced a common challenge for studies using surveillance data on a self-limited disease, possible under-reporting, particularly for mild and subclinical cases [45]. The HFMD incidence used in our analysis is therefore calculated using the incidence of notified cases.

In summary, we investigated the transmission dynamics and mechanism of HFMD in Qinghai and Shanghai, which are typical regions with a large difference in physical and socio-economic environments in Western and Eastern China. HFMD in Qinghai declines over time, but in Shanghai a fluctuated trend is repeatedly present. The potential factors associated with heterogeneities in the spatio-temporal transmission dynamics were analyzed and the inherent mechanisms driving these patterns were captured. Our results suggest that traveling waves are present in both regions, each of which is associated with a different set of significant covariates. The population mobility is characterized by cross-region movement in Qinghai and neighboring movement in Shanghai, respectively. Our findings facilitate the understanding of HFMD transmission mechanisms over geographic regions and may be crucial for assisting the appropriate design of interventions, such as deploying optimal vaccine and changing local driving factors in Huangzhong and Fengxian, to prevent or limit disease spread in these areas.

## Supporting information

S1 Text Supporting text. Supporting text containing details of the spatial SEIR model and the gravity model.
(DOCX)

S1 Fig Local wavelet power spectrum for HFMD cases in each county of Qinghai. (PDF)

S2 Fig Local wavelet power spectrums for HFMD cases in each county of Shanghai. (PDF)

S3 Fig Posterior predictive distribution for HFMD cases in Qinghai. (PDF)

S4 Fig Posterior predictive distribution for HFMD cases in Shanghai. (PDF)

S5 Fig Empirical adjust reproductive number for incidence series of HFMD cases in Qinghai. (PDF)

S6 Fig Empirical adjust reproductive number for incidence series of HFMD cases in Shanghai. (PDF)

S1 Table Bayes factors of model comparison for HFMD cases in Qinghai. (DOCX)

S2 Table Bayes factors of model comparison for HFMD cases in Shanghai. (DOCX)

S3 Table Posterior estimates (mean, 95% credible interval, and median) of the parameters in the dispersion term. (DOCX)
