# Supplementary material for: Transmission center and driving factors of hand, foot, and mouth disease in China: A combined analysis
Source: PLoS Negl Trop Dis. 2020 Mar 9;14(3):e0008070. doi: 10.1371/journal.pntd.0008070 (PMC7062235; doi:10.1371/journal.pntd.0008070)
Supplement: S1 Text — Supporting text containing details of the spatial SEIR model and the gravity model. (DOCX) [file pntd.0008070.s001.docx]

**Supporting Materials for**

**Transmission center and driving factors of Hand, Foot, and Mouth Disease in China: A Combined Analysis**

**Hu et al.**

*Corresponding author. Email: epistat@gmail.com

**The spatial SEIR model**

Epidemic state transitions occur over discrete time *i* (*i*=1,…, t) and discrete space *j* (*j*=1,…, n). The spatial SEIR model gives rise to the following set of difference equations and chain binomial structure:

$$S_{i+1}=S_{i}-E_{i}^{*} E_{ij}^{*}\sim binom\left( S_{ij},\pi_{ij}^{SE} \right)$$

$E_{i+1}=E_{i}-I_{i}^{*}+E_{i}^{*} I_{ij}^{*}\sim binom\left( E_{ij},\pi_{ij}^{EI} \right)$ (1)

$$I_{i+1}=I_{i}-R_{i}^{*}+I_{i}^{*} R_{i}^{*}\sim binom(I_{ij},\pi_{ij}^{IR})$$

$$R_{i+1}=R_{i}-R_{i}^{*}$$

where S, E, I, R denote membership counts in corresponding compartment; E^*^, I^*^, R^*^ denote the number of individuals transiting into corresponding compartment, and $\pi_{ij}^{SE}$, $\pi_{ij}^{EI}$, $\pi_{ij}^{IR}$ denote transition probability from S to E, E to I, I to R, respectively.

The S to E transition, which captures the exposure process, may be expected to vary over space and time. To quantify this process, we assume that each location *j* has an epidemic intensity ($\theta_{j}$) that varies throughout the epidemic. To structure this intensity, each location is associated with a *t* by *p* design matrix $X_{j}$ such that the intensity time series for the location can be calculated as $\theta_{j}=X_{j}\beta^{SE}$ for the shared parameter vector $\beta^{SE}$. This linear predictor prior structure provides an intuitive and flexible lower dimensional representation for the intensity process. The *p* parameters may be used to incorporate any time-varying or invariant covariates affecting the exposure process, such as demographic effects, intervention summaries, and innumerable other spatiotemporal variables, and $\beta^{SE}$ are the corresponding coefficients. To account for movement of individuals in the exposure process, it is necessary to specify the spatial structure of the population under study. A number of $n\times n$ “distance” matrices {$D_{z}:z=1,\ldots,Z$} and associated autocorrelation parameter $\{\rho_{z}\}$ subject to $\sum_{z=1}^{Z} \rho_{z}\leq1$ and {$0{\leq\rho}_{z}<1:z=1,\ldots,z$} are used to build this spatial structure. We choose $z=1$, that is, a single distance metric case in our study. The final parametric form of exposure process is as follows:

$\pi_{ij}^{SE}=1-\exp\left( \left\{ -\eta_{i.}-\sum_{z=1}^{Z} \rho_{z}\left( D_{z}\eta_{i.} \right) \right\}^{h_{i}} \right)$ (2)

where $\eta_{i.}=\{\delta_{i1}e^{\theta_{i1}},\ldots,\delta_{in}e^{\theta_{in}}\}$ ($\delta_{ij}$ and $\theta_{ij}$ denote the proportion of persons who are infectious and the exposure intensity parameter at time *i* in spatial unit *j*, respectively) and $h_{i}$ is the temporal offset capturing the relative length of continuous time over which the events are accumulated. We choose $h_{i}=1$for convenience in our study.

Transitions from E to I, which captures the latent behavior of a pathogen, and I to R, which captures the infectious duration, are considered to be primarily properties of the pathogens, and $\pi_{ij}^{EI}$ and $\pi_{ij}^{IR}$ are therefore unlikely to vary substantially over space. The two quantities are modeled using the exponential compartment membership model of Lekone and Finkenstadt [1] as follows:

$\pi_{ij}^{EI}=1-\exp\left( -\gamma_{EI} \right)$ (3)

$$\pi_{ij}^{IR}=1-\exp\left( -\gamma_{IR} \right)$$

where $\gamma_{EI}$ is the rate at which individuals transition to the infectious from the exposed category and $\gamma_{IR}$ is the rate at which the infectious individuals are recovered/removed. This parameterization corresponds to mean latent and infectious periods ${1/\gamma}_{EI}$ and ${1/\gamma}_{IR}$ respectively.

*Inference*

We apply approximate Bayesian computation (ABC) [2] to the spatial SEIR stochastic epidemic model as the numerous unobserved compartment values cause the parameter space to grow rapidly in the number of location/time points and relatively few parameters are required to simulate such data [3]. For this reason, ABC can be thought of as a dimension reduction strategy for such models. We partition the unknown parameters into the following two components:

${\theta=[\beta^{SE},\gamma}_{EI}{,\gamma}_{IR},\rho]$ (4)

$$\zeta=[S,E, I,R,S^{*},E^{*},I^{*},R^{*}]$$

No matter which compartment, transition matrix, or combination thereof the observed data y (HFMD incidence data) relate to, we may simulate it from a conditional distribution $p(\zeta|\theta)$ using ABC inference. Before doing this, the prior distributions of individual parameters in $\theta$ are specified as follows:

$\beta^{SE}\sim N(0,1)$ (5)

$$\rho\sim beta(1,1)$$

According to the Ministry of Health, People’s Republic of China, the incubation period of HFMD is 3-5 days and the infectious period is about 7-10 days [4]. This indicates a very high chance that an infected individual will become infectious at the beginning of the week after being exposed; a fact that is important given the weekly granularity of the available data. To encode this prior information, we chose to use an exponential transition process, as in Eq. (3), with $\gamma_{EI}$ with a mean of 3.0 and an effective prior sample size of 100. This indicates approximately a 95% chance that an exposed individual will transition to infectious within the first week. Similarly, a mean of 0.8 and effective prior sample size of 100 are chosen for $\gamma_{IR}$, which implies approximately a 55% chance that an infectious individual will transition to recovery within the first week. The prior setting of $\gamma_{EI}$ and $\gamma_{IR}$ can be easily adjusted by comparing the posterior to the established range for the HFMD pathogen.

*The empirically adjusted reproductive number (EA-RN)*

Let the indicator $I_{k}(t_{i},s_{j},s_{l})$ denote the event that a person $k$ from spatial location $s_{j}$ is infected at time $t_{i}$ by contact from within spatial location $s_{l}$, and note that ${P(I}_{k}(t_{i},s_{j},s_{l}))=0$ unless person $k$ is a member of the susceptible class $S$. The expected number of such infections is then:

$E\left[ \sum_{k=0}^{N_{i,j}} {(I}_{k}\left( t_{i},s_{j},s_{l} \right)) \right]=S_{ij}\cdot{P(I}_{k}(t_{i},s_{j},s_{l})|k\epsilon S)$ (6)

and the average per infectious individual at $\left( t_{i},s_{j} \right)$ is $\frac{S_{ij}\cdot{P(I}_{k}\left( t_{i},s_{j},s_{l} \right)\left| k\epsilon S \right)}{I_{ij}}$. In the single distance metric case, the associated contribution to the overall probability $\pi_{ij}^{SE}$

has a simple form:

${P(I}_{k}\left( t_{i},s_{j},s_{l} \right)\left| k\epsilon S \right)=1-exp\{-f(d_{jl})\delta_{il}e^{\theta_{il}}\}$ (7)

The general case, for which we must consider the contribution from each distance metric, can be written as

${P(I}_{k}\left( t_{i},s_{j},s_{l} \right)\left| k\epsilon S \right)=1-exp(-\sum_{z=1}^{Z} \rho_{z}{\{D_{z}\}}_{jl}\cdot\eta_{il})$ (8)

where $\eta_{il}=\delta_{il}e^{\theta_{il}}$.

These expectations can be arranged into a matrix which is a single time unit analogue of the $n\times n$ next generation matrix $G_{jl}(t_{i})$ [5], by averaging the infection counts over the number of responsible infectious individuals. Expressions for the elements, $G_{jl}(t_{i})$, of these matrices are given by premultiplying the expressions for the single and multiple metric cases by the ratio: $\frac{S_{ij}}{I_{ij}}$. The next step in this approach to basic reproductive number estimation is to calculate the dominant eigenvalue of the next generation matrix [5]. However, the row sums of the constructed matrix give the average number of infections caused by each infectious individual in the spatial location with the same index at that time point. To generalize this result to the lifetime of the pathogen, we compute the expected total number of such infections over time:

${R^{(EA)}= m}_{i,j}\sum_{t=t_{i}}^{t_{\infty}} G(t)\cdot\left[ \prod_{k=t_{j+1}}^{t} (1-\pi_{k}^{(IR)}) \right]$ (9)

**The time-series SIR model**

The number of HFMD cases that are locally generated $\pi_{i}(t)$ in county *i* at time *t* is informed by the observed incidences Ii in that district in the preceding weeks, through the serial interval distribution $\omega$ [5]:

$\pi_{i}\left( t \right)=R_{i}(t)\sum_{\tau=1}^{T} \omega\left( \tau\right)I_{i}(t-\tau)$ (10)

where *T* is the cut-off period for the serial interval distribution and $R_{i}(t)$ is the reproduction number in county *i* at time *t* at which secondary cases are observed. The observed weekly incidence in each county are composed of locally infected individuals and infected ones that have moved in from other counties. Hence, the expectation of the observed incidence $I_{i}(t)$ in county *i* at time *t* is a summation of the non-migrating local incidence and the incidence caused by migrants from other counties:

${E(I}_{i}\left( t \right))=\left( 1-f\left( c_{i} \right) \right)\pi_{i}\left( t \right)+\sum_{j\neq i} f\left( c_{i} \right)m_{j, i}\pi_{j}\left( t \right)$ (11)

where $f\left( c_{i} \right)$ denotes fraction of infected individuals in county *i* scattering over other county *j* weighted by a dispersion distribution $m_{j, i}$. The observed incidence $I_{i}\left( t \right)$ is assumed to be Poisson distribution [5]. The dispersion term distributes the migrants over the other districts following a gravity-type model [6]:

$m_{j,i}=\frac{N_{i}^{\alpha}D_{j,i}^{-\delta}}{\sum_{j\neq i} N_{i}^{\alpha}D_{j,i}^{-\delta}}$ (12)

where $m_{j,i}$ denotes migrants from county *j* to county *i*, $N_{i}$ is the population size in county *i*, $D_{j,i}$ is the distance between counties *j* and *i*, and $\alpha$ and $\delta$ denote the dependent effects.

The model parameters are estimated in a Bayesian MCMC analysis. For the reproduction numbers $R_{i}(t)$, a vaguely informative gamma prior is used with mean 1 and shape parameter 2; for the fractions $f\left( c_{i} \right)$ of migrating infected individuals, we assume it to be the same for all counties for simplicity and a beta distribution is used with a mean of 0.1 (Beta(1,9)); for $\alpha$ and $\delta$, uniform priors between -1 and 6 are used, to include the possibility of no population or distance dependence. The analysis is run with 10 chains of length 5000, for each augmented data set; convergence is assessed by eye. The model is analyzed in JAGS using R.

**References**

1 Lekone PE, Finkenstädt BF: Statistical inference in a stochastic epidemic seir model with control intervention: ebola as a case study. Biometrics 2006;62:1170-1177.

2 Rubin D: Bayesianly justifiable and relevant frequency calculations for the applied statistician. Ann Stat 1984;12:1151-1172.

3 Brown GD, Porter AT, Oleson JJ, Hinman JA: Approximate bayesian computation for spatial seir(s) epidemic models. Spat Spatiotemporal Epidemiol 2018;24:27-37.

4 Ministry Of Health PRC: Guideline on preventions and controls of hand-foot-mouth disease of 2008 (in chinese). Capital J Public Health 2008;2:146-148.

5 Cori A, Ferguson NM, Fraser C, Cauchemez S: A new framework and software to estimate time-varying reproduction numbers during epidemics. Am J Epidemiol 2013;178:1505-1512.

6 Zipf G: The p1 p2/d hypothesis: on the intercity movement of persons. Am Sociol Rev 1946:677-689.

**Supporting Information include:**

**S1 Table**. Bayes factors of model comparison for HFMD cases in Qinghai.

**S2 Table**. Bayes factors of model comparison for HFMD cases in Shanghai.

**S1** **Fig**. Local wavelet power spectrum for HFMD cases in each county of Qinghai.

**S2 Fig**. Local wavelet power spectrums for HFMD cases in in each county of Shanghai.

**S3 Fig**. Posterior predictive distribution for HFMD cases in Qinghai.

**S4 Fig**. Posterior predictive distribution for HFMD cases in Shanghai.

**S5 Fig**. Empirical adjust reproductive number for incidence series of HFMD cases in Qinghai.

**S6 Fig**. Empirical adjust reproductive number for incidence series of HFMD cases in Shanghai.
