# Supplementary figures and images for: Transmission center and driving factors of hand, foot, and mouth disease in China: A combined analysis
Source: PLoS Negl Trop Dis. 2020 Mar 9;14(3):e0008070. doi: 10.1371/journal.pntd.0008070 (PMC7062235; doi:10.1371/journal.pntd.0008070)

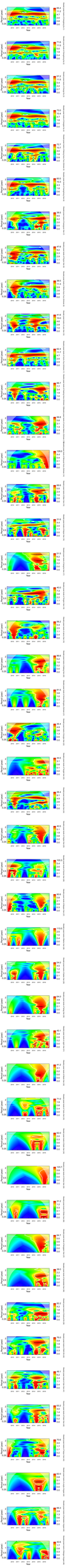

Supplement: S1 Fig — (PDF) [file pntd.0008070.s002.pdf]

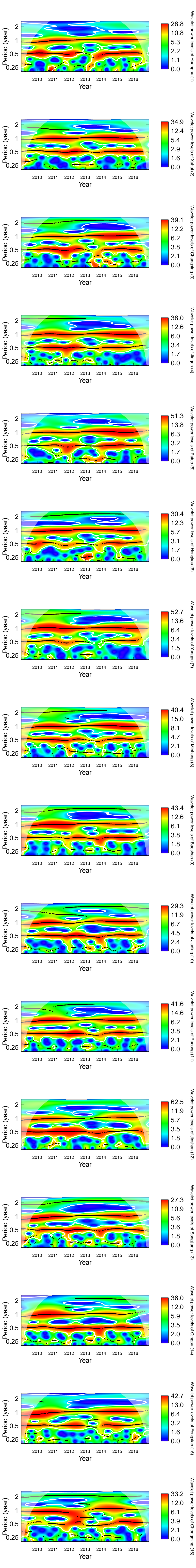

Supplement: S2 Fig — (PDF) [file pntd.0008070.s003.pdf]

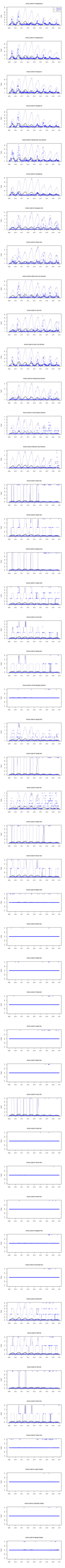

Supplement: S3 Fig — (PDF) [file pntd.0008070.s004.pdf]

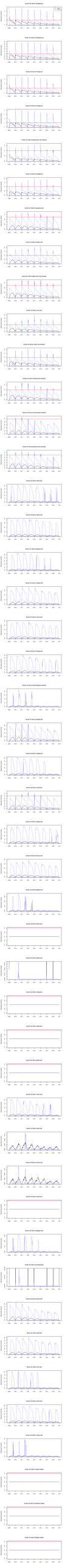

Supplement: S5 Fig — (PDF) [file pntd.0008070.s006.pdf]
