# Supplementary material for: Transmission center and driving factors of hand, foot, and mouth disease in China: A combined analysis
Source: PLoS Negl Trop Dis. 2020 Mar 9;14(3):e0008070. doi: 10.1371/journal.pntd.0008070 (PMC7062235; doi:10.1371/journal.pntd.0008070)

CAR model for Huangpu

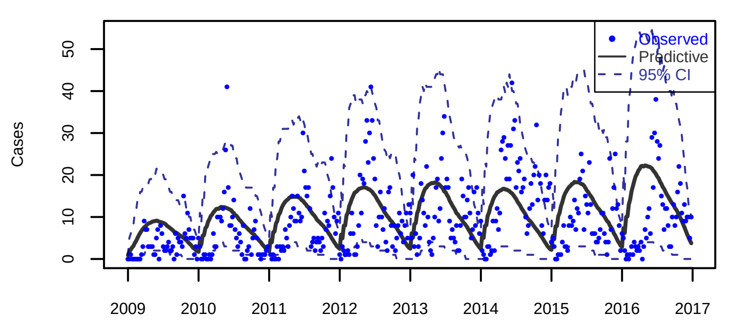

CAR model for Xuhui

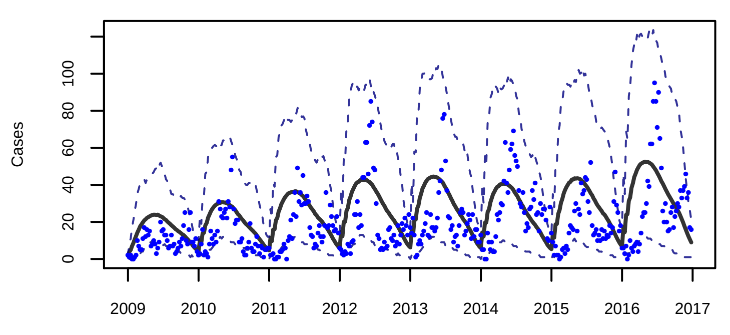

CAR model for Changning

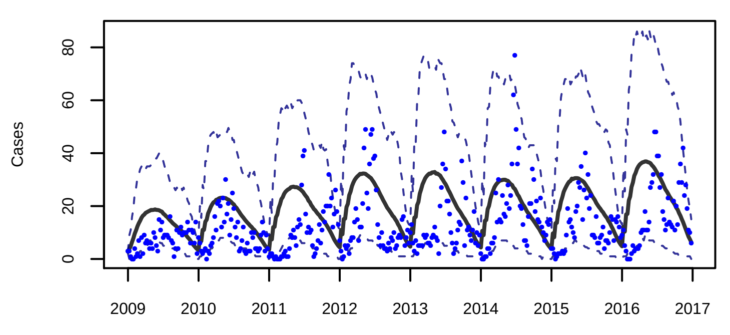

CAR model for Jingan

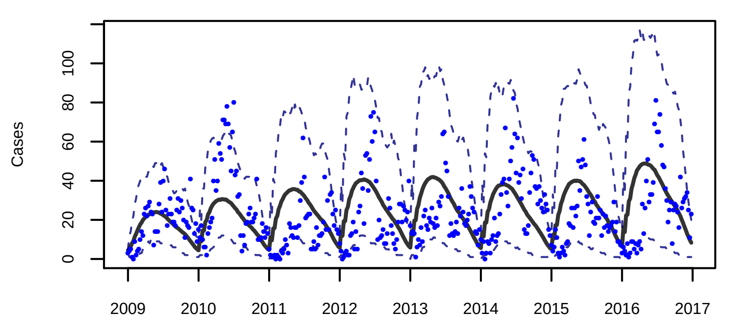

CAR model for Putuo

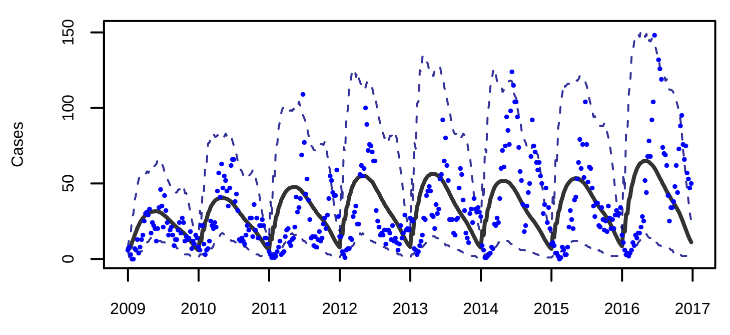

CAR model for Hongkou

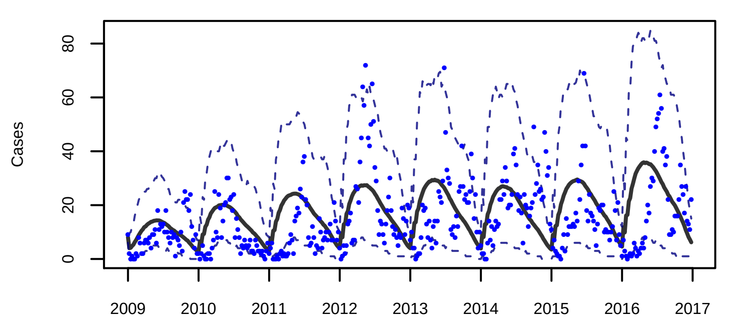

CAR model for Yangpu

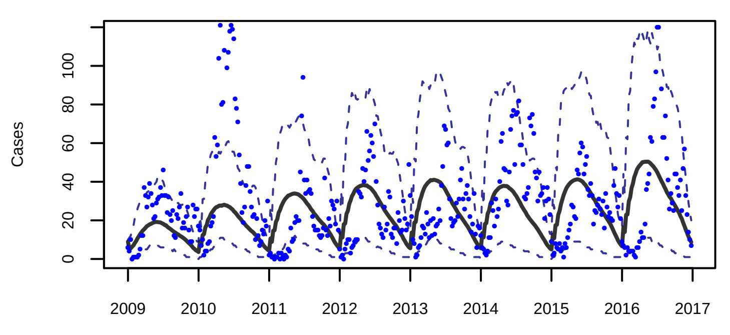

CAR model for Minhang

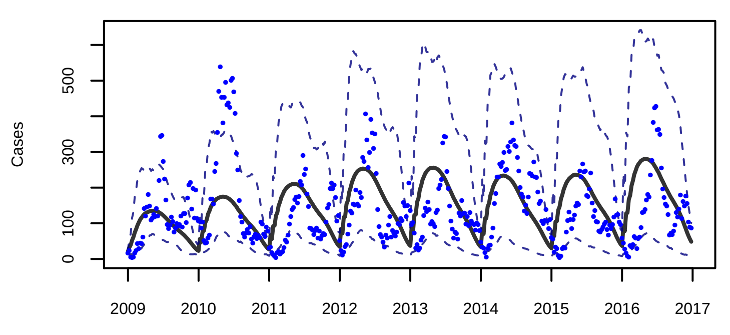

CAR model for Baoshan

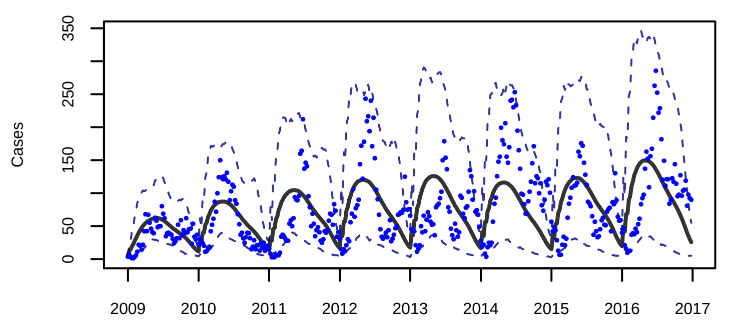

CAR model for Jiading

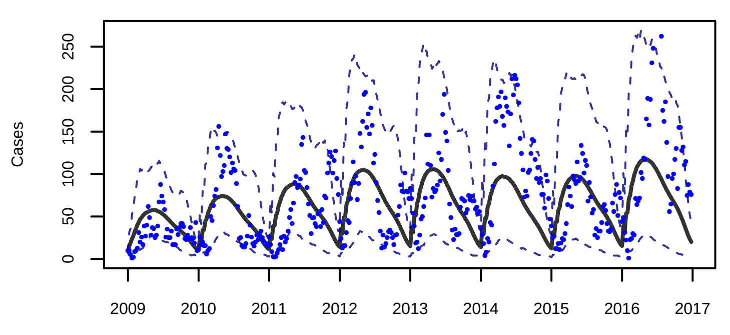

CAR model for Pudong

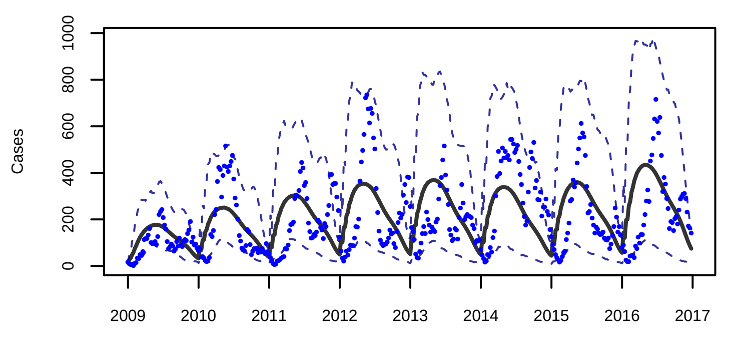

CAR model for Jinshan

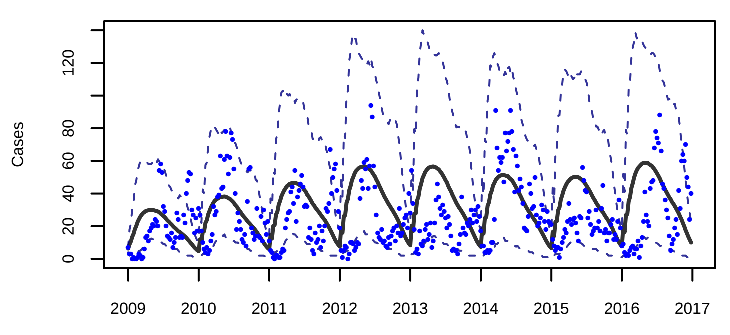

CAR model for Songjiang

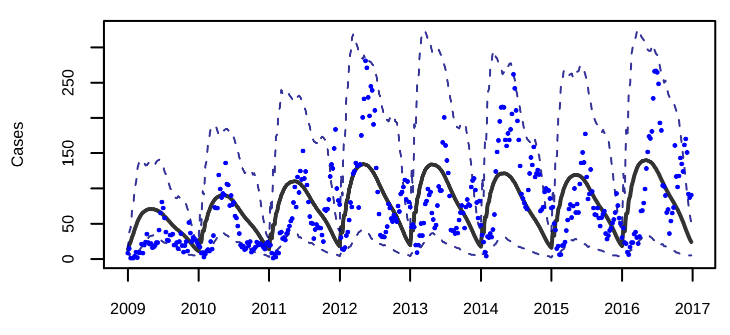

CAR model for Qingpu

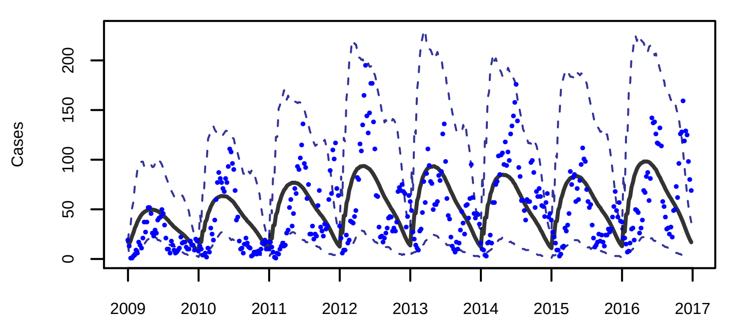

CAR model for Fengxian

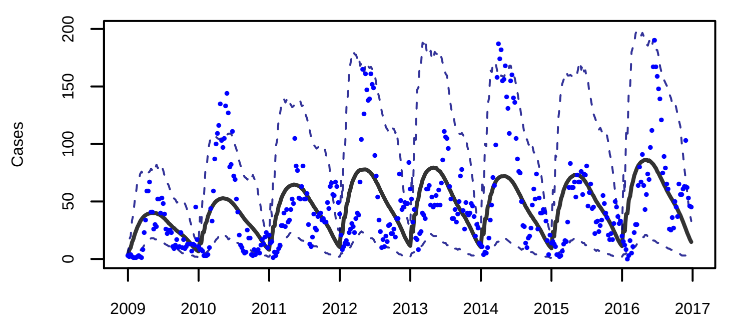

CAR model for Chongming

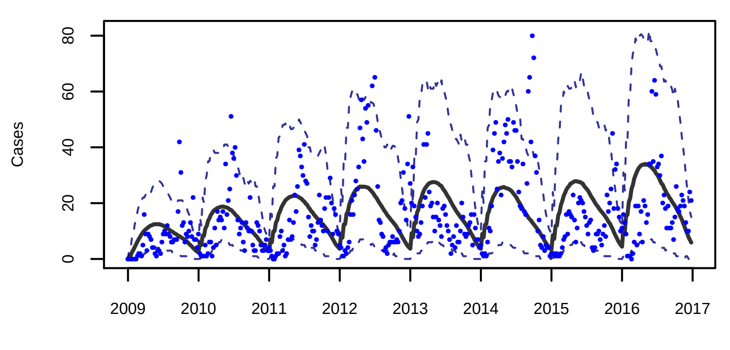

Supplement: S4 Fig — (PDF) [file pntd.0008070.s005.pdf]
