# Supplementary material for: Transmission center and driving factors of hand, foot, and mouth disease in China: A combined analysis
Source: PLoS Negl Trop Dis. 2020 Mar 9;14(3):e0008070. doi: 10.1371/journal.pntd.0008070 (PMC7062235; doi:10.1371/journal.pntd.0008070)
Supplement: S6 Fig — (PDF) [file pntd.0008070.s007.pdf]

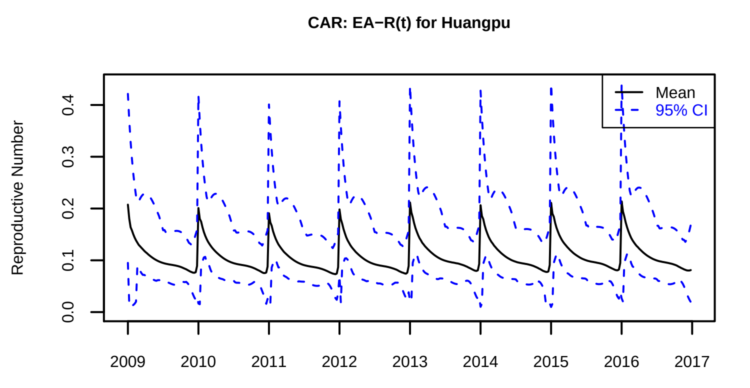
CAR: EA−R(t) for Huangpu
Mean
95% CI
Reproductive Number
2009
2010
2011
2012
2013
2014
2015
2016
2017

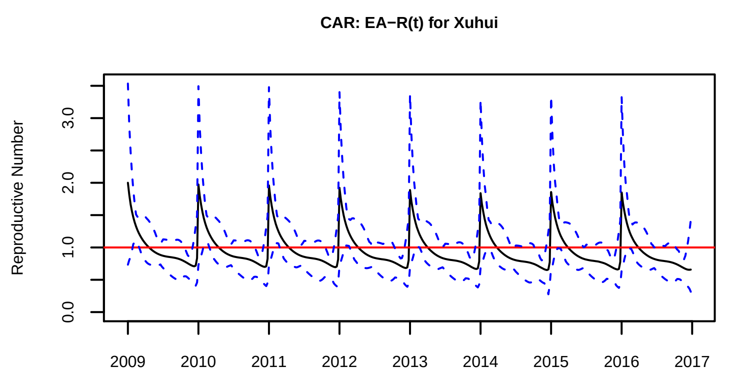
CAR: EA−R(t) for Xuhui
Reproductive Number
2009
2010
2011
2012
2013
2014
2015
2016
2017

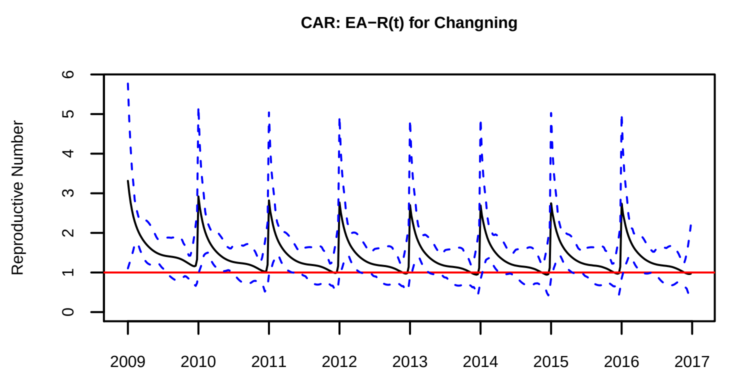
CAR: EA−R(t) for Changning
Reproductive Number
2009
2010
2011
2012
2013
2014
2015
2016
2017

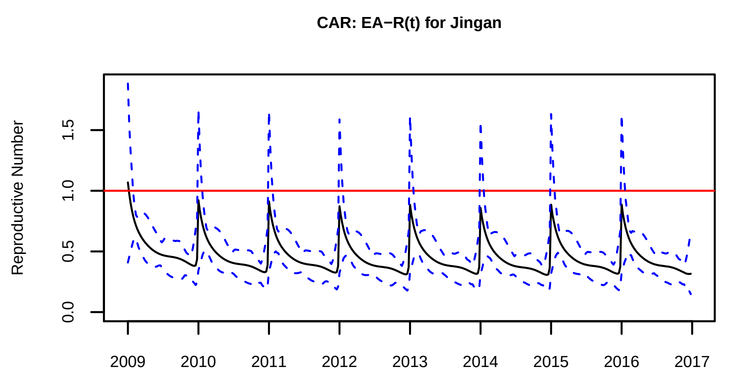
CAR: EA−R(t) for Jingan
Reproductive Number
2009
2010
2011
2012
2013
2014
2015
2016
2017

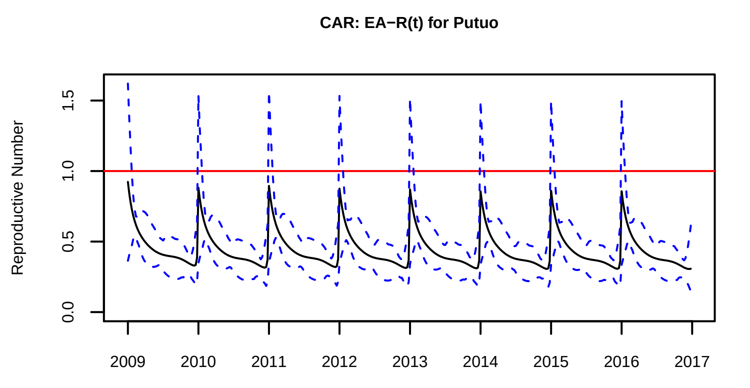
CAR: EA−R(t) for Putuo
Reproductive Number
2009
2010
2011
2012
2013
2014
2015
2016
2017

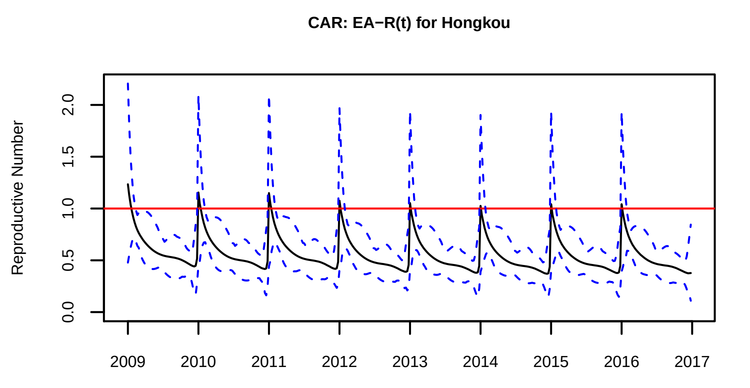
CAR: EA−R(t) for Hongkou
Reproductive Number
2009
2010
2011
2012
2013
2014
2015
2016
2017

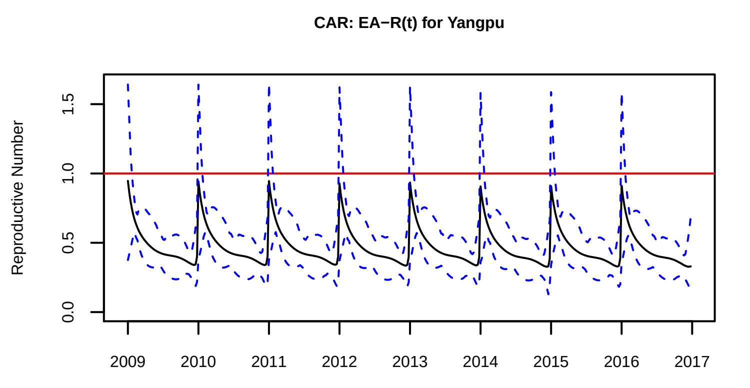
CAR: EA−R(t) for Yangpu
Reproductive Number
2009
2010
2011
2012
2013
2014
2015
2016
2017

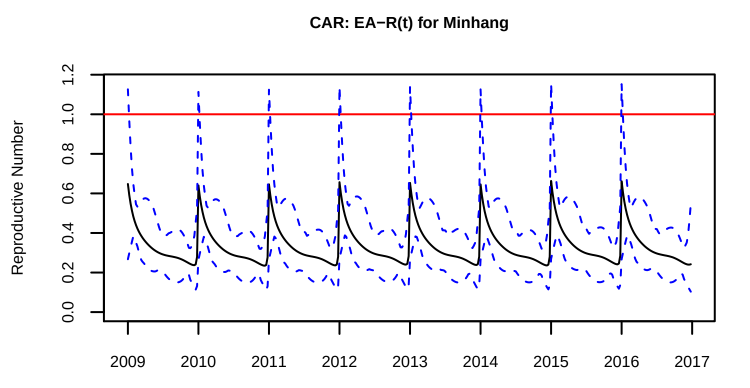
CAR: EA−R(t) for Minhang
Reproductive Number
2009
2010
2011
2012
2013
2014
2015
2016
2017

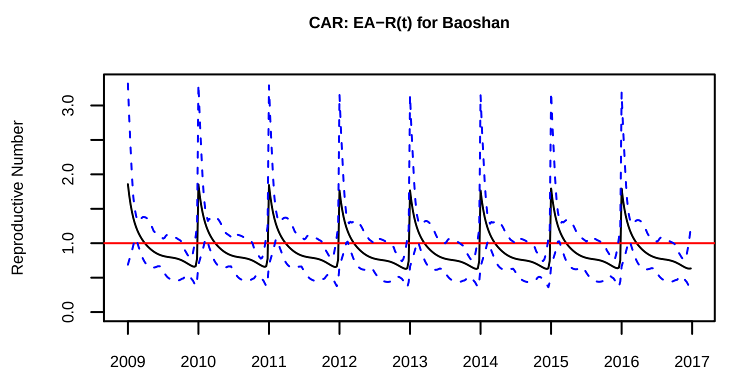
CAR: EA−R(t) for Baoshan
Reproductive Number
2009
2010
2011
2012
2013
2014
2015
2016
2017

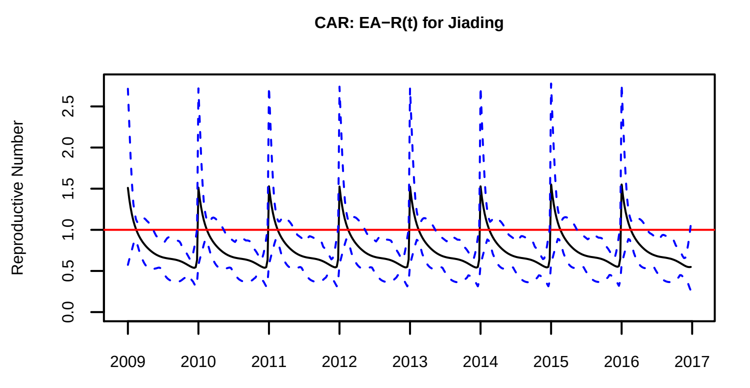
CAR: EA−R(t) for Jiading
Reproductive Number
2009
2010
2011
2012
2013
2014
2015
2016
2017

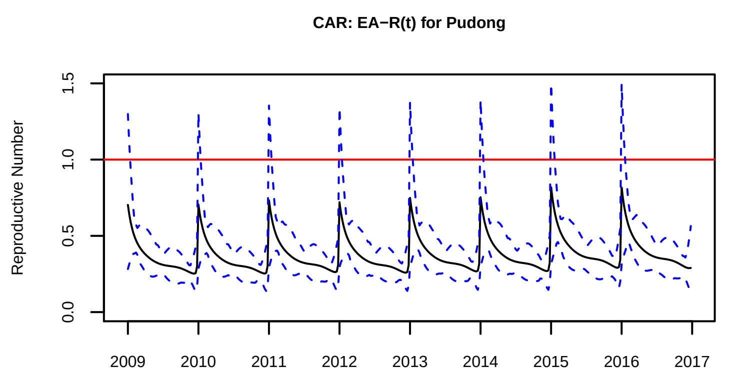
CAR: EA−R(t) for Pudong
Reproductive Number
2009
2010
2011
2012
2013
2014
2015
2016
2017

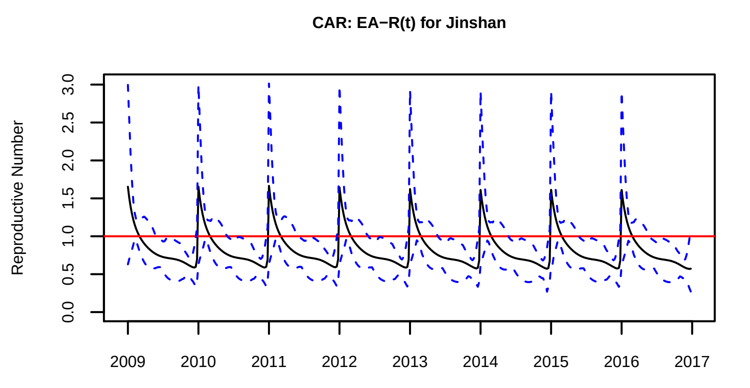
CAR: EA−R(t) for Jinshan
Reproductive Number
2009
2010
2011
2012
2013
2014
2015
2016
2017

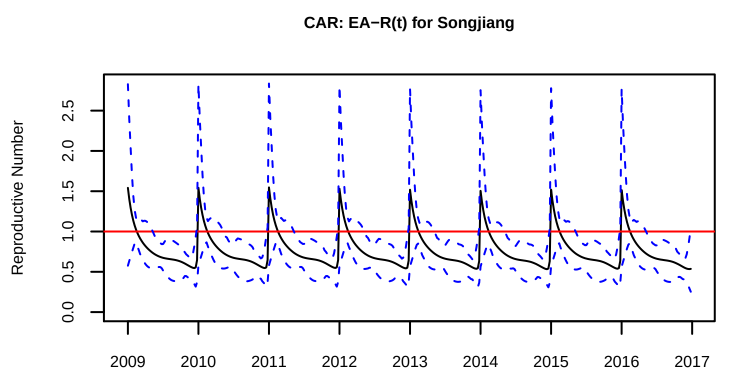
CAR: EA−R(t) for Songjiang
Reproductive Number
2009
2010
2011
2012
2013
2014
2015
2016
2017

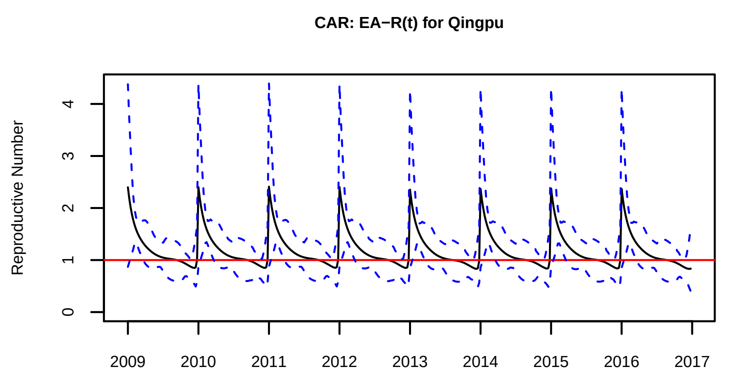
CAR: EA−R(t) for Qingpu
Reproductive Number
2009
2010
2011
2012
2013
2014
2015
2016
2017

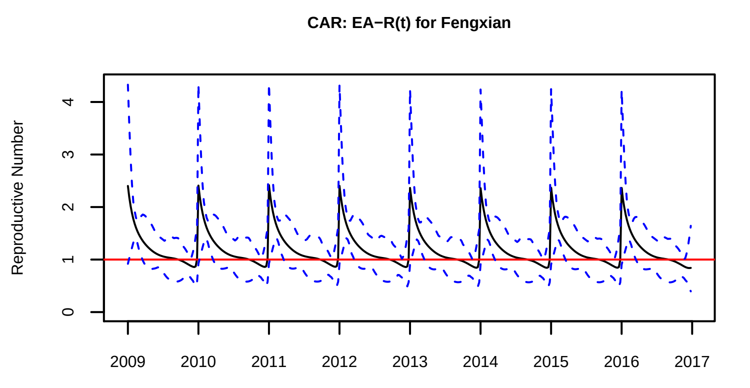
CAR: EA−R(t) for Fengxian
Reproductive Number
2009
2010
2011
2012
2013
2014
2015
2016
2017

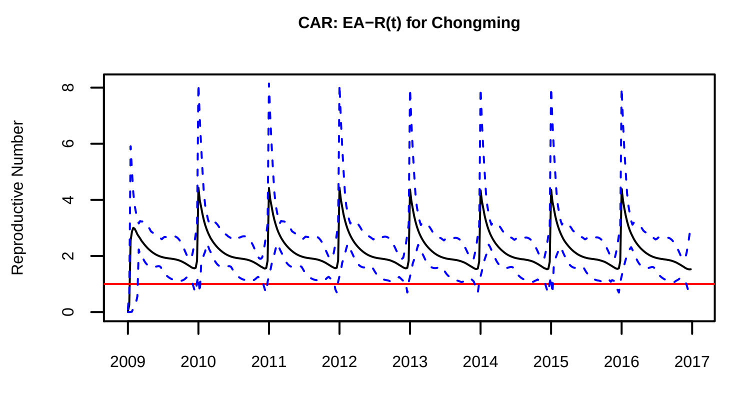
CAR: EA−R(t) for Chongming
Reproductive Number
2009
2010
2011
2012
2013
2014
2015
2016
2017
