# Supplementary material for: Transmission center and driving factors of hand, foot, and mouth disease in China: A combined analysis
Source: PLoS Negl Trop Dis. 2020 Mar 9;14(3):e0008070. doi: 10.1371/journal.pntd.0008070 (PMC7062235; doi:10.1371/journal.pntd.0008070)
Supplement: S1 Table — (DOCX) [file pntd.0008070.s008.docx]

**S1 Table. Bayes factors of model comparison for HFMD cases in Qinghai**

|  | M1 | M2 | M3 | M4 | M5 | M6 |
| --- | --- | --- | --- | --- | --- | --- |
| M1 | 1 | 0.289 | 0 | 0 | 0 | 0 |
| M2 | 3.456 | 1 | 0 | 0 | 0 | 0 |
| M3 | Inf | Inf | 1 | 0.034 | 0 | 0 |
| M4 | Inf | Inf | 29.3 | 1 | 0 | 0 |
| M5 | Inf | Inf | Inf | Inf | 1 | 0.013 |
| M6 | Inf | Inf | Inf | Inf | 75 | 1 |

Bayes factor = likelihood probability of model in ROW / that of model in COLUMN. For example, 3.456 = M2/M1. As this value is over 1, it is believed that M2 is more strongly supported by the data under consideration than M1.
