# Supplementary material for: Transmission center and driving factors of hand, foot, and mouth disease in China: A combined analysis
Source: PLoS Negl Trop Dis. 2020 Mar 9;14(3):e0008070. doi: 10.1371/journal.pntd.0008070 (PMC7062235; doi:10.1371/journal.pntd.0008070)
Supplement: S3 Table — (DOCX) [file pntd.0008070.s010.docx]

**S3 Table. Posterior estimates (mean, 95% credible interval, and median) of the parameters in the dispersion term.**

| Parameters | Mean | Q_0.025_ | Q_0.975_ | Median |
| --- | --- | --- | --- | --- |
| $\alpha$ | 0.640 | 0.557 | 0.721 | 0.640 |
| $\delta$ | 1.170 | 1.082 | 1.257 | 1.170 |
